# Spring-mediated cranioplasty for scaphocephaly: techniques and outcomes

**DOI:** 10.1007/s00381-026-07300-1

**Published:** 2026-06-11

**Authors:** Maura R. Guyler, David P. Perrault, Kirin Naidu, Santiago Lopez-Becerra, Jordan W. Swanson, Scott P. Bartlett, Jesse A. Taylor, Sameer Shakir

**Affiliations:** Division of Plastic, Reconstructive, and Oral Surgery, Children’s Hospital of Philadelphia, 3401 Civic Center Blvd, Philadelphia, PA 19104 USA

**Keywords:** Sagittal craniosynostosis, Spring-mediated cranioplasty, Surgical outcomes, Cranial springs, Minimally invasive strip craniectomy

## Abstract

**Purpose:**

Spring-mediated cranioplasty (SMC) is an established minimally invasive technique for the treatment of sagittal craniosynostosis. This study describes our surgical technique and evaluates long-term morphometric outcomes following SMC.

**Methods:**

A retrospective review was performed of subjects who underwent SMC as primary treatment of sagittal craniosynostosis at our institution between 2011 and 2019. Demographic variables, perioperative data, spring characteristics, and cranial index (CI) were obtained. Subjects were stratified by their use of post-operative helmeting. Longitudinal CI changes were evaluated using linear mixed-effects models. Differences in CI change between subjects receiving 2 vs 3 springs were compared using Mann–Whitney *U* tests. Linear regression was used to evaluate total spring force as a predictor of percent change in CI.

**Results:**

Seventy subjects were included. Seventy-four percent (*n* = 52) of the cohort received springs treatment only, whereas 26% (*n* = 18) additionally underwent post-operative helmeting. Mean pre-operative CI was 0.67. Mean age at spring placement was 3.5 months and mean time to spring removal was 108 days. The mean total spring force independent of spring number, measured to 20 mm initial opening, was 26.3 N. Mean post-operative CI at >10 years was 0.76 and 0.74 for non-helmeted and post-operative helmeted subjects, respectively. There was no significant difference in change in CI between subjects who received 2 or 3 springs, despite a significantly different mean total spring force (18.6 N versus 27.9, *p* < 0.001). Linear regression demonstrated that different minimum spring forces were required to reach adequate CI improvement based on total spring number.

**Conclusions:**

Spring-mediated cranioplasty is associated with durable correction of sagittal craniosynostosis with sustained long-term improvement as measured by cranial index. Further evidence is needed to elucidate predictors of CI change when using SMC, its effects on regional head shape changes in three dimensions, and to further explore the role of adjunct therapies such as helmeting.

## Introduction

Sagittal craniosynostosis is the most prevalent form of single-suture craniosynostosis. Surgical options range from minimally invasive procedures, such as strip craniectomy with post-operative helmeting and spring-mediated cranioplasty (SMC), to open cranial vault remodeling [1–3]. Many institutions have shifted toward minimally invasive procedures due to the favorable perioperative complication profile and equivalent morphological outcomes [2]. Specifically, these minimally invasive procedures are associated with decreased soft tissue, bony, and dural dissection leading to decreased blood loss and hospital stay and may preserve adjacent cranial suture growth centers, affecting subsequent cranial growth [4–7].

Use of cranial springs as a distraction device in preclinical studies was initially introduced by Persing et al. [8]. Lauritzen et al. then clinically adapted the technique and published an outcomes study of the first 100 cases, further validating the technique [9, 10]. In this technique, cranial springs are placed across a craniectomy site to primarily widen the biparietal diameter and secondarily limit the frontooccipital compensatory growth over time [11–14]. In Lauritzen’s cohort, CI improved on average from 0.67 to 0.74 across 6 months [9]. There were no documented deaths or serious complications [9].

SMC allows for gradual expansion, often resulting in equivocal aesthetic outcomes when compared to strip craniectomy with post-operative helmeting therapy [11–14]. This technique has been adopted by several centers globally and serves as the primary technique utilized at our institution for the treatment of sagittal craniosynostosis before 6 months of age [15]. More recently, we have begun to evaluate the interplay between SMC and a limited period of post-operative helmeting for the treatment of severe scaphocephaly.

Despite its stated advantages, one critical question of this technique remains the durability of its functional and morphometric head shape improvements at the completion of skull growth [16]. Shakir et al. previously evaluated the mid-term outcomes of SMC for sagittal craniosynostosis, focusing on the stability of head shape correction as measured by cranial index (CI) through 5 years post-operatively [15]. We sought to build on previously published long-term cohort studies regarding outcomes of spring-mediated cranioplasty and objectively evaluate our evolving technique and outcomes [17]. We hypothesized that SMC is associated with a safe, durable, and effective functional and aesthetic head shape correction for the primary treatment of sagittal craniosynostosis.

## Methodology

A retrospective cohort study was performed on subjects who underwent primary SMC at the Children’s Hospital of Philadelphia prior to the age of 6 months between August 2011 and November 2019. Inclusion criteria included subjects with head computed tomography (CT) scans, multiplanar radiographs, and/or morphometric measurements of CI throughout the study period. Subjects were stratified by post-operative helmeting status. Additional exclusion criteria included syndromic synostosis, history of prior cranial vault remodeling, incomplete imaging, and/or inadequate follow-up.

Demographic and perioperative variables collected included sex, race, post-operative helmeting status, duration of helmeting, pre-operative CI, and age at spring placement and removal. Surgical variables collected included EBL, pre-operative and post-operative hemoglobin, transfusion requirement, length of stay, and procedure duration. Spring-specific data were collected for each case and included the total number of springs, total Newton force (measured to a 20-mm opening), and individual anterior, middle, and posterior forces. Complications were categorized using the Clavien–Dindo classification system. Reoperation data was also collected including indication, timing, CI at reoperation, and post-operative complications from the reoperation. Primary outcomes included cranial index (CI) and percent change in CI (%ΔCI). The CI was defined as the ratio of maximal biparietal width to maximal occipitofrontal length, multiplied by 100. Percent change in CI was calculated as:

$$\%\Delta\mathrm{CI}=\lbrack\left({\mathrm{CI}}_{\mathrm{Final}}-{\mathrm{CI}}_{\mathrm{Initial}}\right)/{\mathrm{CI}}_{\mathrm{Initial}}\rbrack\times100$$

Descriptive statistics were used to summarize the data.

Data analyses were performed with R 4.5.1. Longitudinal changes in CI following SMC were analyzed using linear mixed-effects models (LMMs). The primary outcome was percentage change in CI from the pre-operative baseline. Outliers were identified for post-operative days 1–3 using the interquartile range (IQR) method, defined as values exceeding Q3 + 1.5 × IQR or below Q1 − 1.5 × IQR. Mixed-effects models were specified as: Percent Change ~ Time Point + (1 | Subject ID). Time point was modeled as a categorical fixed effect, and subject-specific random intercepts were included to account for within-subject correlational cross repeated measures. Estimated marginal means (EMMs) were computed for each time point, and one-sample *t*-tests were used to evaluate whether mean percentage changes significantly differed from baseline. Differences between pre- and post-operative CI in subjects who received 2 versus 3 springs were assessed using a Mann–Whitney *U* test.

We additionally sought to identify the minimum spring force required for subjects who received 7+ years of follow-up to achieve adequate clinical improvement from baseline. In our study, this was defined as ≥9% change in CI, similar to the pooled mean 10.4% change in the existing literature [18]. This analysis included subjects who underwent SMC only and was further stratified by spring number. Pearson correlation was used to assess the relationship between total spring force and percent change in CI for this cohort. Simple linear regression was used to evaluate total spring force as a predictor of percent change in CI. These analyses were then stratified by spring number. Regression outputs and equations were used to calculate the predicted spring force needed to achieve a 9% increase in CI.

### Technique

Our surgical technique has been documented previously [18–20]. Springs are custom fabricated in-house. Stainless steel wire (0.053″ or 0.059″—wire diameter) is cut to a length of 8 to 15 cm and then bent into a “U” shape. A three-pronged plate bender is used to create angular footplates. The force is measured in N by a tensiometer set at a distance of 20 mm to simulate the strip of craniectomy width. Intraoperatively, the patient is placed on a cerebellar. The incision pattern has evolved slowly and depends on the ability to perform the surgery effectively through an increasingly smaller scalp incision. Initially, two incisions were made, one near bregma and one near lambda. More recently, a single Chevron incision located at the mid-parietal level is utilized. If needed later, the incision can be incorporated into a conventional bicoronal incision. Dissection is carried down to the subgaleal plane, and a tunnel is made between the anterior and posterior fontanelles. A 10–15-mm sagittal suturectomy is typically performed. The springs are axially bent to match the skull curvature in the sagittal plane. Two to three springs are typically placed—the springs are now placed further away (20–25 mm) from the coronal and lambdoid sutures to limit the potential for asymmetric elevation of the parietal plates across the patent sutures. Following the placement of the anterior and posterior spring, a mid-parietal spring may be placed if severe scaphocephaly or a saddle deformity is present. The sagittal craniectomy bone is then morcellated and used as a bone graft across the suturectomy and spring footplate sites to minimize bony defects and provide a barrier between the dura and hardware during removal. Spring position is verified by radiographs obtained within 24 h post-operatively. Based on surgeon discretion, parental desires, and phenotypic severity, subjects may undergo post-operative helmeting [20]. Subjects undergo a second outpatient procedure for hardware removal, 3–4 months after the index operation [18, 20].

## Results

In total, *n* = 70 subjects met inclusion. The majority were male (77%, *n* = 54) and Caucasian (84%, *n* = 59) (Table 1). A minority of the cohort underwent post-operative helmeting (26%, *n* = 18). For subjects undergoing post-operative helmeting, the mean duration was 5.08 months, with a range of 2.5–10 months.

**Table 1:** Demographic—summary of demographic variables among our cohort who underwent SMC for sagittal craniosynostosis

*N*	70
Sex	
Male	54 (77%)
Female	16 (23%)
Race	
Caucasian	59 (84%)
Black or African American	6 (9%)
Other	5 (7%)
Post-op helmeting	
Yes	13 (26%)
No	52 (74%)
Average duration of helmeting (months)	5.08 (range: 2.5–10)

### Index operation

Mean age at index operation was 3.5 months, and the mean pre-operative CI value was 0.67. The mean estimated blood loss (EBL) for spring placement surgery was 60 mL. Seven percent (*n* = 5) of subjects required an intraoperative blood transfusion. Three subjects required a post-operative blood transfusion. The mean operative time for spring placement was 141 min, and mean length of stay was 1.79 days (Table 2).

**Table 2:** Spring placement surgical data—summary of intraoperative and perioperative parameters during spring-mediated cranioplasty

Average age at spring placement	3.5 (range: 2.8–4.6 months)
Average pre-operative cranial index (CI)	0.67 (range: 0.58–0.76)
Average estimated blood loss—spring placement	60 (range: 5–250 mL)
Blood transfusion—spring placement	5 (7%)
Average pre-operative hemoglobin—spring placement	11.27 (range: 9.1–13.2)
Average post-operative hemoglobin—spring placement	9.1 (range: 6.3–12.3)
Average procedure length—spring placement	141 (range: 48–284 min)
Average length of stay—spring placement	1.79 (range: 1–5 days)

Most (*n* = 57, 81%) had three springs placed, whereas 13 subjects (19%) received two springs. The mean pre-operative CIs were similar between groups. Subjects who had 2 springs had a mean pre-operative CI of 0.72, whereas subjects with 3 springs had a mean pre-operative CI of 0.68 (*p* = 0.06). The mean spring force for all subjects measured at a 20-mm opening was 26.3 N. The mean spring force for subjects who received two springs was 18.6, compared to subjects who received 3 springs with a mean force of 28.1 N (*p* < 0.001). The median spring force for subjects who received three springs was 28.1 N. Force distribution differed based on spring positioning, with heavier spring forces disproportionately utilized posteriorly (Table 3).

**Table 3:** Spring characteristics—summary of the spring variables and characteristics utilized as part of our spring-mediated cranioplasty

Spring number	
2	13 (19%)
3	57 (81%)
Pre-operative mean CI	
2 springs	0.72 (range: 0.65–0.78)
3 springs	0.68 (range: 0.58–0.81)
Force (N)—measured to 20 mm opening	Mean: 26.27
Total median—2 springs	19.6 (IQR: 17.4–20.2)
Anterior	9.1 (IQR: 8–9.8)
Posterior	10.2 (IQR: 9.6–10.7)
Total median—3 springs	28.1 (IQR: 25–30.9)
Anterior	8.9 (IQR: 8.05–9.8)
Middle	9.3 (IQR: 8.3–10.5)
Posterior	9.7 (IQR: 8.5–10.8)

Mann–Whitney *U* tests revealed no statistically significant difference in the change in CI between subjects who received 2 or 3 springs (*p* = 0.63) (Table 4).

**Table 4:** Spring number and change in CI—summary of the median change in CI between pre- and post-operative CI based on spring number

Spring number	*n*	Median %ΔCI
2 springs	8	13.4%
3 springs	33	13.1%

### Spring removal

Subjects underwent spring removal on average at 108 days, and the mean age at spring removal was 7.2 months. The mean EBL for the spring removal was 19 mL, and no subjects required an intraoperative blood transfusion. The mean procedure length was 85.4 min, and the length of hospital stay was 0.39 days (Table 5).

**Table 5:** Spring removal surgical data—summary of intraoperative and perioperative parameters during spring removal surgery

Average time in springs (days)	108 (range: 68–161 days)
Average age at spring removal	7.2 (range: 5.4–13.6 months)
Average estimated blood loss—spring removal	19 (range: 5–150 mL)
Blood transfusion—spring removal	0 (0%)
Average pre-operative hemoglobin—spring removal	11.7 (8.4–14.4)
Average procedure length—spring removal	85.37 min (range: 36–148)
Average length of stay—spring removal	0.39 days (range: 0–2 days)

### Complications and revisions

The overall post-operative complication rate was 7%. Complications were classified using the Clavien–Dindo system. The two grade I events included hardware migration and a seroma. One grade II complication was observed—a cerebrospinal fluid (CSF) leak. Two grade IIIb complications, a pseudomeningocele repair and hardware migration, warranted reoperation. No grade IIIa complications were noted (Table 6).

**Table 6:** Surgical complications—summary of post-operative complications following SMC at our institution, categorized by Clavien–Dindo grade

Clavien–Dindo grade	Count	Complication(s)
I	2	Hardware migration (×1), seroma
II	1	Cerebrospinal fluid (CSF) leak
IIIa	0	None
IIIb	2	Pseudomeningocele repair (×1), operative hardware migration
Total	5	All complications

Five subjects (7%) required secondary cranial expansion following primary spring-mediated cranioplasty. Indications included a persistent occipital bullet (*n* = 1), scaphocephaly (*n* = 2), and concerns for elevated intracranial pressure (*n* = 2). The mean pre-operative CI for subjects undergoing revision was 0.69 with a range of 0.65–0.74. The mean post-operative CI prior to revision surgery was 0.71 with a range of 0.66–0.77. Revision procedures include posterior vault reconstruction (*n* = 3), frontal orbital expansion (*n* = 1), and total cranial vault reconstruction (*n* = 1) (Table 7).

**Table 7:** Reoperation data—summary and indications of reoperation following primary SMC

Pre-op CI (initial)	Pre-op CI (revision)	Indication	Reoperation procedure
0.68	0.68	Persistent occipital bullet	Posterior vault reconstruction
0.74	0.71	Persistent scaphocephaly	Fronto-orbital expansion with cranial vault remodeling
0.65	0.66	Persistent scaphocephaly	Posterior vault reconstruction
0.68	0.77	Concern for elevated ICP: papilledema on eye exam	Posterior vault reconstruction
–	–	Concern for elevated ICP: head-banging and headaches	Total cranial vault reconstruction

### Long-term morphometric outcomes

**Fig. 1:**
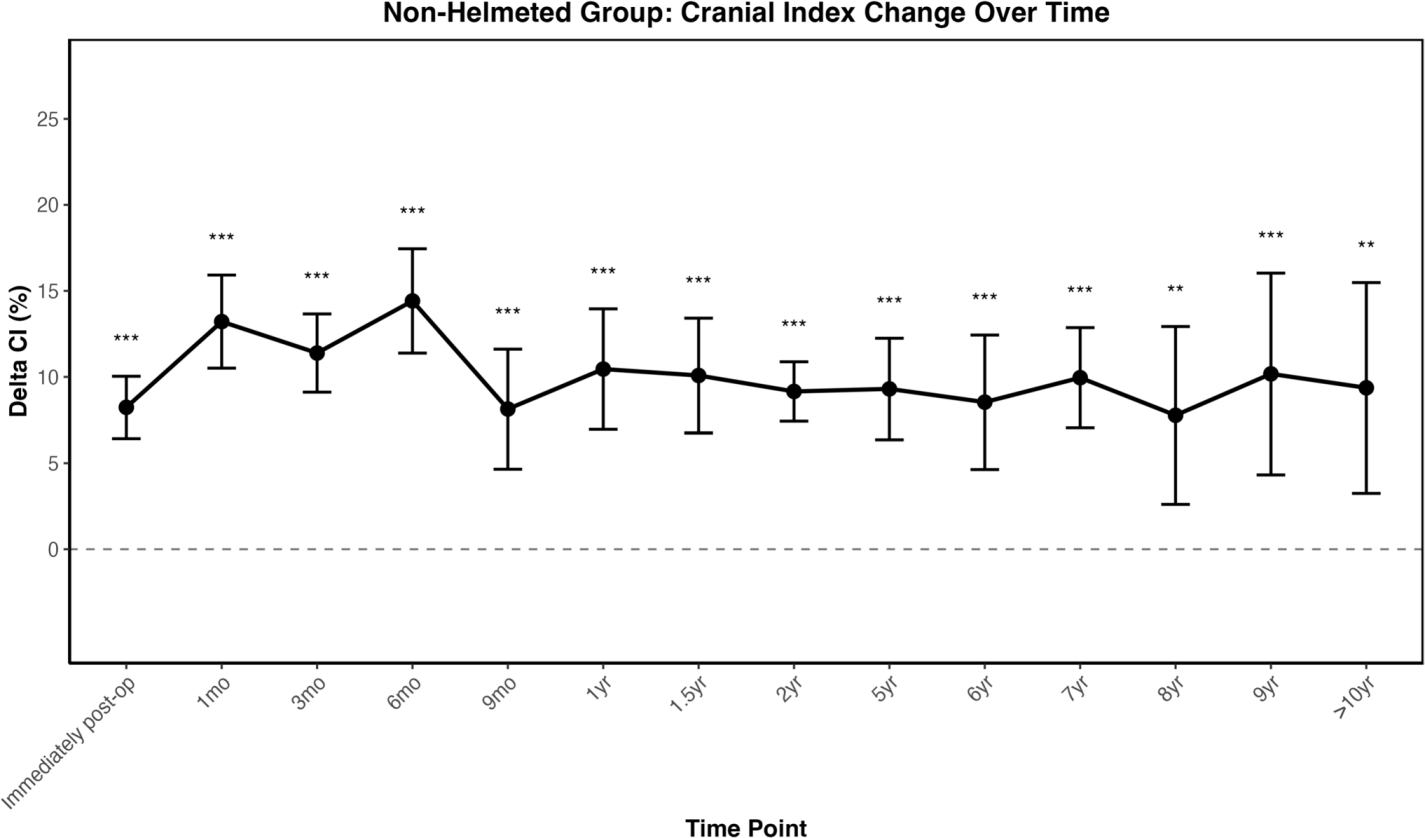
Non-helmet group: cranial index change over time: this figure demonstrates the mean percent change from pre-operative levels at each measured time point for subjects who received post-operative helmeting only (*n* = 49, 234 data points). Asterisks represent the degree of statistical significance from baseline (* *p* < 0.05, ** *p* < 0.01. *** *p* < 0.005), represented by the dashed line

In subjects undergoing SMC alone, there was a statistically significant improvement in percent change in CI from baseline. Additionally, CI was corrected to a normative range (0.75–0.80) and remained normal despite small changes over time. Linear mixed effect modeling revealed a mean 8.23% increase (absolute CI: 0.74) in the immediate post-operative period (post-operative days 1–3). Peak improvement of 14.42% increase (absolute CI: 0.79) occurred at 6 months. There was a subsequent slowing of improvement at 9 months to 8.13% (absolute CI: 0.75), which subsequently rebounded at 1-year follow-up to 10.45% increase (absolute CI: 0.77). The lowest mean percentage increase of CI was 7.77% (absolute CI: 0.75), seen at 8 years post-operatively. Long term, subjects at 10+ years of follow-up had a mean increase in pre-operative CI to 9.36% (Fig. 1, Table 8). The mean CI for subjects with >8 years of follow-up was 0.75 and the mean CI for subjects with >10 years of follow-up was 0.76.

**Table 8:** EMM percent change in cranial index (CI) at each post-operative time point following spring-mediated cranioplasty in non-helmeted subjects. Values represent mean percent change from baseline with standard error, 95% confidence intervals, and *p* values from one-sample *t*-tests evaluating each point to baseline

Time point	Mean CI	Mean % change	SE	95% CI	*p* value
Immediately post-op	0.74	8.23	0.91	[6.43, 10.04]	<0.001
1 mo	0.79	13.21	1.37	[10.51, 15.92]	<0.001
3 mo	0.77	11.39	1.15	[9.11, 13.66]	<0.001
6 mo	0.79	14.42	1.54	[11.38, 17.45]	<0.001
9 mo	0.75	8.13	1.77	[4.65, 11.61]	<0.001
1 yr	0.77	10.45	1.78	[6.95, 13.95]	<0.001
1.5 yr	0.76	10.08	1.69	[6.75, 13.41]	<0.001
2 yr	0.76	9.15	0.87	[7.43, 10.88]	<0.001
5 yr	0.75	9.3	1.49	[6.36, 12.25]	<0.001
6 yr	0.76	8.53	1.98	[4.63, 12.43]	<0.001
7 yr	0.75	9.95	1.48	[7.04, 12.86]	<0.001
8 yr	0.75	7.77	2.62	[2.61, 12.93]	0.00333
9 yr	0.75	10.18	2.97	[4.32, 16.03]	<0.001
>10 yr	0.76	9.36	3.11	[3.25, 15.48]	0.00285

**Fig. 2:**
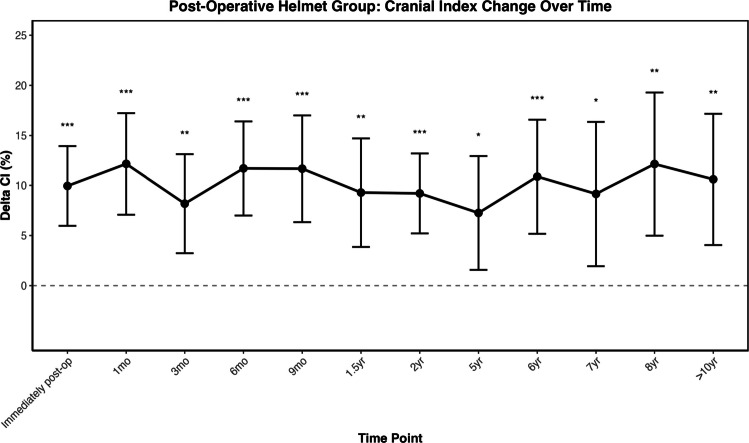
Post-operative helmeting group: cranial index change over time: this figure demonstrates the mean percent change from pre-operative levels at each measured time point for subjects who received post-operative helmeting (*n* = 15, 90 data points). Asterisks represent degree of statistical significance from baseline (* *p* < 0.05, ** *p* < 0.01. *** *p* < 0.005), represented by the dashed line

In subjects undergoing SMC and post-operative helmeting, there was a statistically significant improvement in percent change in CI from baseline. Similar to the non-helmeted group, the CI was corrected to a normal value and remained normal at 10+ years. Linear mixed effect modeling revealed a mean 9.94% increase in CI (absolute CI: 0.73) in the immediate post-operative period (post-operative days 1–3). Peak improvement of 12.15% increase in CI (absolute CI: 0.74) occurred at 1 month post-operatively. After >8 years of follow-up, the mean CI was 0.72 and mean CI for >10 years was 0.74 (Fig. 2, Table 9).

**Table 9:** EMM percent change in cranial index (CI) at each post-operative time point following spring-mediated cranioplasty in subjects who underwent post-operative helmeting. Values represent mean percent change from baseline with standard error, 95% confidence intervals, and *p* values from one-sample *t*-tests evaluating each point to baseline

Time point	Mean CI	Mean % change	SE	95% CI	*p* value
Immediately post-op	0.73	9.94	1.97	[5.97, 13.92]	<0.001
1 mo	0.74	12.15	2.55	[7.07, 17.23]	<0.001
3 mo	0.73	8.17	2.47	[3.23, 13.11]	0.00154
6 mo	0.75	11.7	2.35	[6.99, 16.4]	<0.001
9 mo	0.74	11.67	2.68	[6.33, 17]	<0.001
1.5 yr	0.67	9.28	2.73	[3.86, 14.71]	0.00104
2 yr	0.73	9.2	1.97	[5.21, 13.19]	<0.001
5 yr	0.72	7.25	2.86	[1.57, 12.93]	0.01293
6 yr	0.72	10.87	2.87	[5.17, 16.58]	<0.001
7 yr	0.74	9.15	3.63	[1.94, 16.36]	0.01342
8 yr	0.72	12.14	3.6	[4.98, 19.29]	0.00108
>10 yr	0.74	10.61	3.3	[4.05, 17.17]	0.00181

### Minimum spring force

**Fig. 3:**
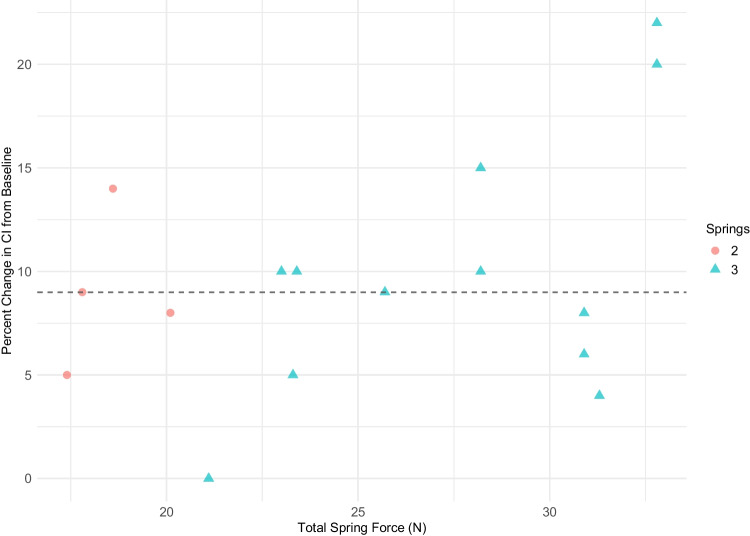
Relationship between total spring force and percent change in cranial index (CI) from baseline in subjects with sagittal craniosynostosis at >8 years following spring-mediated cranioplasty. Each point represents an individual patient, differentiated by number of springs placed (2 vs 3). The dashed horizontal line indicates the 9% improvement threshold

A total of 16 subjects had follow-up data >7 years and were included in this analysis. Four subjects received 2 springs, and 12 subjects received 3 springs. Nine out of 16 (56%) achieved ≥9% improvement in CI. When stratified by spring number, the minimum force at which ≥50% of subjects achieved ≥9% improvement was 17.4 N for subjects who received 2 springs and 21.1 N for subjects who received 3 springs (Fig. 3).

## Discussion

We sought to objectively evaluate the long-term outcomes of subjects who underwent SMC for the primary treatment of sagittal craniosynostosis. We hypothesized that the technique is associated with an aesthetic and functional head shape correction over the long term. These data suggest that SMC is associated with a durable correction over the long term with limited aesthetic and functional relapse. Across our 10+ years of follow-up, subjects maintained significant morphometric improvement as measured by cranial index (CI) from the pre-operative state with a low complication rate.

Subjects demonstrated significant improvement in the early post-operative period and then sustained correction in the long term. At 10+ years of follow-up, the magnitude of correction was 10.61% and 9.36% for subjects who underwent post-operative helmeting and those who only underwent SMC, respectively, demonstrating durability of SMC. Regarding helmeting, there does not appear to be a long-term benefit to CI. However, CI is not a comprehensive index for a successful aesthetic outcome after surgery, which has been previously discussed [21]. Although helmeting does not benefit CI, this data should not undermine the utility of post-operative helmeting. For example, Nguyen et al., using 3D photogrammetry, observed that there are regional changes in head shape after helmet therapy not captured by CI [22]. There has yet to be a long-term comparison of SMC with and without helmeting that could provide insight into this question.

The findings from our cohort support the previously published reports of stable long-term outcomes. Previously published CI improvements ranged from a 6.0% increase to a 21.6% increase [9, 11, 14, 23–25]. David et al. reported +7.6% CI change at 12 years and Ng et al. reported stable CI at 9 years [17, 26]. The growth trajectory we observed mostly aligned with the reports by Shakir et al. and Ng et al. [15, 26]. In their cohorts, CI rapidly increased in the immediate post-operative period and plateaued at 3 months post-operatively [15, 26]. However, for subjects who did not receive helmeting, there was an uptick of growth in the early post-operative period followed by a peak in growth at 6 months. In contrast, growth in helmeted subjects peaked earlier at 1 month. The increase in CI after SMC peaks earlier in helmeted subjects; however, this does not appear to be meaningful in the long term.

Our analysis revealed no significant difference in change in CI between subjects who received 2 or 3 cranial springs. This is consistent with previously published literature [27]. It is interesting that subjects who received 2 springs required 8 fewer Newtons to achieve a 9% increase in CI than subjects who received 3 springs. This finding highlights that the biomechanical properties of the viscoelastic skull likely play a large and undescribed role. This observation highlights the importance of the surgeon’s assessment. Recently published early work aims to describe the biomechanical nature of the skull-spring relationship in a preclinical finite element analysis model [28]. Once these variables can be better quantified, our understanding of spring forces should further evolve.

Our data remain insufficient to determine the minimum Newton force that would predict an effective change in cranial index. There is likely a threshold for total Newton force that must be employed to overcome the viscoelastic nature of the skull and lead to the desired expansion. Spring force has been previously correlated with increased change in CI [20]. However, despite numerous reports on the relationship of spring characteristics on CI and head shape, the lowest effective force needed remains an elusive goal [28–32].

Comparing SMC to open vault procedures warrants discussion. Outcomes like cranial index change and intracranial volume have been found to be mostly equivalent between open and minimally invasive surgical techniques [33–35]. A meta-analysis of 26 studies of sagittal craniosynostosis revealed equivocal aesthetic outcomes, when assessed subjectively and objectively, between techniques [36]. These findings were reaffirmed in a meta-analysis of 15 studies with 1436 subjects by Duan and Yang which demonstrated similar post-operative results between minimally invasive and open cohorts, with a cranial index range of 0.70 to 0.79 [37]. A cohort study evaluating head shape using 3D photography and metrics such as the frontal bossing index (FBI), occipital bullet index, vertex narrowing index, and scaphocephalic index found results suggesting improved longevity of head shape correction in subjects who underwent spring-mediated cranioplasty compared to cranial vault remodeling [38].

SMC demonstrates a favorable safety profile [11, 17, 39–41]. Compared to open cranial vault remodeling, SMC is associated with significantly lower blood loss, transfusion requirements, length of ICU stay, hospital stay, and fewer complications [11, 23, 38, 42–46]. The reported major adverse events of SMC include sagittal sinus tears, hematomas, hemorrhage, displacement of hardware, exposure, and infection [26, 40, 47, 48, 48]. In our cohort, 5 complications (7%) were noted, but no major adverse events occurred. There was one pseudomeningocele, which was successfully repaired. Secondary operations were performed to correct the aesthetic head shape in three subjects and functional concerns in another two subjects. There is concern for under-correction resulting in persistent scaphocephaly or concern for elevated ICP; however, this is a minimal risk in our experience. SMC may also be advantageous by reducing anesthesia exposure by decreasing operative times, which could impact neurocognitive development in young children [39].

There are several limitations to the current study. The retrospective design and the natural attrition that occurs with long-term follow-up may introduce some selection bias into the outcomes. Stratification by helmeting subgroup reduced the statistical power of the analysis; however, this was necessary since helmeting is a distinct and impactful treatment that may have changed the outcomes. Our analysis of minimum spring force was performed in an effort to establish the lowest effective force required to achieve a positive outcome; however, we currently do not have the sample size or range of spring forces to make this determination. Post-operative imaging was limited by institutional efforts to minimize radiation exposure in the pediatric population; therefore, longitudinal CI data were derived from cephalograms and standardized clinical measurements, which may introduce measurement variability [23]. Additionally, changes in CI were our primary outcome measure, and using CI as the sole measurement is not sufficient to fully characterize the morphometric outcomes after operative management of sagittal craniosynostosis; however, it is a simple and accessible metric to report [21].

## Conclusion

Spring-mediated cranioplasty is a safe and effective technique to address the morphometric and functional concerns associated with sagittal craniosynostosis. Our cohort study demonstrates that SMC corrects CI to a normal range, which is stable at 10 years of follow-up. Furthermore, the rate of complications and reoperation for functional or aesthetic concerns is low. Further research is needed to greater elucidate the predictive factors for cranial head shape in spring-mediated cranioplasty and the impact of adjunct treatments.

## Data Availability

The datasets generated during and/or analyzed during the current study are available from corresponding author on reasonable request.
